# Health workers readiness and practice in malaria case detection and appropriate treatment: a meta-analysis and meta-regression

**DOI:** 10.1186/s12936-021-03954-1

**Published:** 2021-10-24

**Authors:** Hosein Azizi, Reza Majdzadeh, Ayat Ahmadi, Elham Davtalab Esmaeili, Behrouz Naghili, Mohammad Ali Mansournia

**Affiliations:** 1 Department of Epidemiology and Biostatistics, School of Public Health, Tehran University of Medical Sciences, Tehran, Iran; 2 School of Public Health, Knowledge Utilization Research Center, and Community Based Participatory Research Center, Tehran University of Medical Sciences, Tehran, Iran; 3 Knowledge Utilization Research Center, Tehran University of Medical Sciences, Tehran, Iran; 4 Research Center of Psychiatry and Behavioral Sciences, Tabriz University of Medical Sciences, Tabriz, Iran; 5 Road Traffic Injury Research Center, Tabriz University of Medical Sciences, Tabriz, Iran; 6 Infectious and Tropical Diseases Research Center, Tabriz University of Medical Sciences, Tabriz, Iran

**Keywords:** Health worker, Malaria, Appropriate treatment, Practice, Meta-regression

## Abstract

**Background:**

Health workers (HWs) appropriate malaria case management includes early detection and prompt treatment with appropriate anti-malarial drugs. Subsequently, HWs readiness and practice are considered authentic evidence to measure the health system performance regarding malaria control programme milestones and to issue malaria elimination certification. There is no comprehensive evidence based on meta-analysis, to measure the performance of HWs in case management of malaria. This study aimed to evaluate HWs performance in early malaria case detection (testing) and the appropriate treatment.

**Methods:**

The published literature in English was systematically searched from Medline, Scopus, Embase, and Malaria Journal up to 30th December 2020. The inclusion criteria were any studies that assessed HWs practice in early case detection by malaria testing and appropriate treatment. Eligibility assessment of records was performed independently in a blinded, standardized way by two reviewers. Pooled prevalence estimates were stratified by HWs cadre type. Meta-regression analysis was performed to explore the impact of the appropriateness of the method and risk of bias as potential sources of the heterogeneity in the presence of effective factors.

**Results:**

The study pooled data of 9245 HWs obtained from 15 included studies. No study has been found in eliminating settings. The pooled estimate for appropriate malaria treatment and malaria testing were 60%; 95% CI: 53–67% and 57%; 95% CI: 49–65%, respectively. In the final multivariable meta-regression, HWs cadre and numbers, appropriateness of study methods, malaria morbidity and mortality, total admissions of malaria suspected cases, gross domestic product, availability of anti-malarial drugs, and year of the publication were explained 85 and 83% of the total variance between studies and potential sources of the heterogeneity for malaria testing and treating, respectively.

**Conclusion:**

HWs adherence to appropriate malaria case management guidelines were generally low while no study has been found in eliminating countries. Studies with the inappropriateness methods and risk of bias could be overestimating the actual proportion of malaria appropriate testing and treating. Strategies that focus on improving readiness and early identification of acute febrile diseases especially in the countries that progress to malaria elimination should be highly promoted.

## Background

Globally, there were an estimated 229 million malaria cases in 2019 in 87 malaria endemic countries, declining from 238 million in 2000. Malaria case incidence (i.e. cases per 1000 population at risk) reduced from 80 in 2000 to 58 in 2015 and 57 in 2019 globally. Between 2000 and 2015, global malaria case incidence declined by 27%, and between 2015 and 2019 it declined by less than 2%, indicating a slowing of the rate of decline since 2015. However, malaria is a significant health problem in around 100 tropical, subtropical, and temperate countries [1, 2].

Appropriate case management of malaria patients includes early case detection and prompt treatment with appropriate anti-malarial drugs. It can prevent severe disease and fatal outcome [3]. Furthermore, effective case-management of malaria contains early identification of malaria infection from febrile suspected cases, access to diagnostic tests, especially rapid diagnostic tests (RDTs) or microscopy, and getting first-line anti-malarial drugs where adherence to diagnosis and treatment guidelines by health workers (HWs), and also patient compliance to medication, are essential [4].

Early malaria case detection from suspected cases compatible with suspected malaria is an initial key in the appropriate malaria case management process without considering positive or negative test results. It has been shown that early malaria detection (testing) among suspected malaria cases or febrile disease in any transmission setting is an effective strategy to prevent the fatal outcome, missing cases, and under-diagnosis in malaria control/elimination programme [5]. The World Health Organization (WHO) recommends that any malaria suspected case in any transmission setting should be tested by either RDT or microscopy [6, 7]. Subsequently, HWs readiness and practice is considered as an authentic evidence to measure the performance regarding malaria control programme milestones and to issue malaria elimination certification [8]. However, evidence has shown that HWs practice in malaria case management varies in different settings [9]. More importantly, in low transmission areas and countries in the elimination phase, appropriate malaria case management by HWs and system readiness would decrease due to the low episodes of malaria [8].

There is no comprehensive evidence based on systematic reviews, to measure the performance of HWs in case management of malaria patients [7]. The first objective of this systematic review and meta-analysis was the pooled proportion of malaria testing (early case detection) from febrile cases compatible with suspected malaria, and the second objective is to evaluate the pooled prevalence estimate of appropriate malaria treatment by HWs and decomposing the potential sources of the heterogeneity. Therefore, this study was performed to gather and synthesize evidence of HWs performance and management of suspected and confirmed malaria cases.

## Methods

### Search strategy

The published literature in English was systematically searched from databases including Medline via Ovid, Scopus, Embase, Malaria Journal, up to 30th December 2020. Grey literature also was explored from WHO and CDC reports, congress papers, and records. The study searched articles reporting on appropriate case management of confirmed or suspected malaria cases at any age presenting to HWs of any providers in all transmission settings.

The search used both free text words and medical subject headings (MeSH terms). The initial search terms were “malaria” OR “fever” in the title and/or abstract. The final search used the relevant MeSH terms and text words related to malaria or febrile disease case management in conjunction with “malaria” OR “fever” AND “provider” OR “worker” OR “system” AND “management” OR “readiness” OR “practice” OR “performance” OR “identify” OR “diagnosis” OR “treat”. The reference lists of the retrieved studies were also screened with the purpose to identify other potential data sources.

### Eligibility criteria

The inclusion criteria were any studies that assessed HWs routine practice or performance (included testing and treating, at least) in malaria case management or febrile patients compatible with suspected malaria.

Exclusion criteria included studies conducted for active case finding and/or population screening, studies evaluated only HWs knowledge and attitude, studies performed for assessing effects of any particular intervention on malaria case management; reviews, letters, conference abstracts, editorials, commentaries, and qualitative studies were also excluded if they reported incomplete data on malaria case management.

### Data selection and extraction

Eligibility assessment of records was performed independently in a blinded, standardized way by two reviewers (HA, EDE). First, the title and abstract were screened, and the two reviewers screened and selected relevant full-text papers. Quantitative and qualitative data (with brief description) were extracted based on the pre-specified criteria into an excel sheet (HA).

Extracted data included the year of publication, name of author, Country, the cadre of HWs, number of HWs, demographic characteristics of patients and HWs, number of febrile or suspected malaria cases, the type of malaria diagnostic tests including RDT or microscopy for suspected cases, proportion of malaria testing, availability of RDT and anti-malarial drugs, number of malaria confirmed patients, proportion of appropriate treatment for confirmed patients, malaria incidence (per 1000 at-risk), malaria mortality (per 100,000), national population size, and Gross domestic product (GDP) per capita.

### Quality assessment

The quality and strength of the included articles measured using Crombie’s tool [10]. Crombie’s instrument comprises seven items for quality assessment of cross-sectional studies including sample size and representativeness of the samples, appropriateness of methods, ascertaining of data and outcome variables (malaria testing and appropriate treatment), reliability and validity of the measurements, clarity and non-respondents report, appropriateness of statistical methods and adequacy of the analyses. The quality scoring ranged from 0 to 7 for each article. The final included studies were decided through the consensus of the two authors (HA, EDE). If disagreements, the third author (BN) would make the final decision.

### Risk of bias assessment

Due to the variety of included studies from various malaria transmission setting and population, the risk of bias was assessed based on parameters of Newcastle–Ottawa Scale [11] and authors consensus discussion. The following parameters were considered for risk of bias assessment: sampling method and strategy (using random and unbiased sampling methods), adequate sample size, using appropriate data collection methods (for example assessed HWs practice by valid and reliable methods including role-playing, or direct view, performed blood samples of the patients, interviews with HWs, and/or having a gold standard for evaluating HWs practice), adequacy of response rate, inclusion/exclusion criteria, sample representativeness, and appropriate statistical analysis.

The final scoring system comprised 11 criteria of rating different risk of bias elements for each eligible article out of 12 scores. Scale weights (unbiased sampling and data collection method had highest weights) were recommended by authors for each parameter of the scoring system, as proposed in other meta-analyses. Studies were classified into three levels of risk of bias: low risk (9–12 points), moderate risk (5–8 points), and high risk (< 5 points) (Table 1).

### Criteria for HWs performance and appropriate malaria case management

Appropriate malaria case management by HWs was defined as the early case detection by malaria testing from febrile patients compatible with suspected malaria, and the appropriate treatment of confirmed malaria cases [6].

The *primary outcome* measure was the proportion of malaria testing (RDT or microscopy) from febrile cases compatible with suspected malaria; the *secondary outcome* was the proportion of appropriate first-line anti-malarial drug prescription. The study also aimed to explore and decomposing the potential sources of heterogeneity in the pooled prevalence estimates of malaria case detection (testing) and appropriate treatment.

Appropriate treatment was defined as malaria parasite-positive cases treated with the recommended dosage first-line anti-malarial drugs (*not only prescribed any antimalarial drugs*) in the particular artemisinin-based combination therapy (ACT). Conducting diagnostic tests (RDT or microscopy) from suspected cases by HWs was used as malaria testing proportion.

### Statistical analysis

The pooled estimate and 95% Confidence interval (CI) were calculated for proportions of malaria testing, and appropriate treatment of confirmed cases. Pooled estimates were stratified by HWs cadre (Medical doctor “(MD)”, “Non-MD”, “Non-MD and MD”) to evaluate the effect of HWs cadre on the proportion of malaria testing and malaria appropriate treatment. Multivariable meta-regression analysis was performed to explore the impact of the appropriateness of the method and risk of bias, potential variables and sources of the heterogeneity after retaining significant heterogeneity in the subgroup analysis, using a back-step procedure and deleting unassociated variables every step, to find significant retained variables in the regression model. STATA version 13 (Stata Corp, College Station, TX, USA) was used for meta-analysis.

## Results

### Study selection and characteristics

**Fig. 1:**
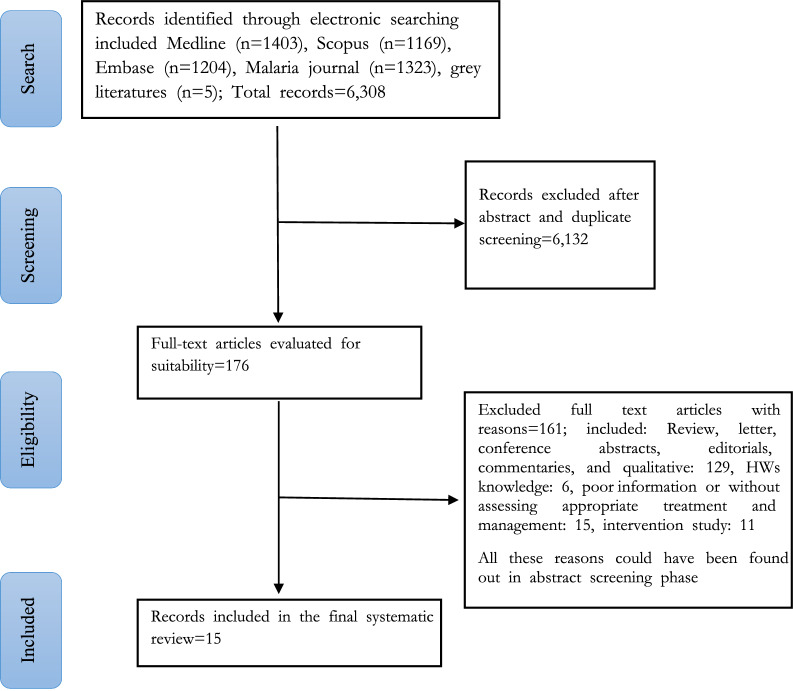
Search flow diagram

Overall, a total of 6308 records were retrieved, after searching for possible relevant studies. Of those, 6132 studies were removed due to duplicate and abstract screening and 176 studies were eligible and their full-text assessed for final inclusion. Finally, 15 articles were included in the systematic review, meta-analysis, and meta-regression for assessing appropriate malaria case management and the pooled estimates in the proportions of malaria testing and appropriate treatment by HWs (Fig. 1).

Table 1 demonstrates characteristics of studies included by considering national population, GDP, malaria morbidity and mortality measures. All fifteen eligible studies were cross-sectional designs in which data were retrieved from HWs, health facilities, or medical records. Studies were published between 2009 and 2020 and eleven (73.3%) studies were conducted in Africa, one study was conducted in each of the countries included India, Haiti, Papua New Guinea, and Vanuatu. It is noteworthy that there were no eligible studies conducted from countries in the elimination phase.

Studies included various types of HWs cadres including Community health workers (CHWs), medical doctors (MD), clinicians and medical assistance, nurses and nurse aid, drug stores and pharmacies, attendant, and midwives. Overall, HWs categorize into three groups including “Non-MD”, “MD and Non-MD”, and “MD” based on primary studies reporting. A total of 9245 HWs and 7004 Health facilities (HFs) have participated in included studies. Out of those, three and one of the studies were not reported the number of HFs and HWs in their studies, respectively. Almost, 51% of HWs were female. Gender of provider was not reported in six studies. The mean age (35.6 years) of HWs ranged from 29 to 40 (Table 2).

HWs proportion who trained for malaria diagnostic and treatment at least once in the last year was reported in eight final studies. The highest trained proportion was 90% (Plucinski et al. [12] and the lowest was 40% (Zurovac et al. [13]). Out of 7004 HFs in included studies, the overall proportion of availability of malaria diagnostic tests (RDT or microscopy) was 65%. Likewise, the overall percentage in availability of the first-line anti-malarial drugs proportion was 82.5%. The highest proportion of anti-malarial drugs availability was 95% in the study by Gallay and colleagues [14] and the lowest was 58% in the study by Aguemon et al. [15]. However, 5 studies, and 7 studies not reported availability of anti-malarial drugs, and availability of diagnostics tests, respectively (Table 2).

Table 3 shows the number of malaria patients and appropriate case management by HWs. A total of 46,574 admissions, 39,322 malaria suspected cases (febrile disease and/or epidemiological history, sign and symptoms compatible with malaria), and 11,256 confirmed malaria cases were reported among final eligible studies. Among final eligible articles only one study [16] did not report the absolute number of confirmed malaria cases. Out of 11,256 malaria patients, 57% were females. The age distribution of confirmed malaria patients was reported in 7 studies. More than 60% of malaria patients were ≥ 5 years and the majority of patients was lower than 15 years.

**Table 1:** Characteristics of studies included by malaria transmission setting and risk of bias assessment

First author	Year	Country(s)	Population (million)	GDP per capita (1000 USD)	Study type	Malaria incidence*	Malaria mortality**	Risk of Bias
Abiodun [31]	2020	Nigeria	210.5	2.23	Cross-sectional	296.08	32.77	Low risk
Cohen [22]	2020	sub-Saharan	1,100	1.59	Cross-sectional	180	33	Low risk
Garg [34]	2020	India	1,338.6	2.09	Cross-sectional	18.6	1.89	High risk
Aguemon [15]	2018	Benin	11.17	1.22	Cross-sectional	293.7	41.16	Low risk
Bonful [18]	2019	Ghana	29.12	0.59	Cross-sectional	266.4	48.11	High risk
Worges [17]	2019	Zambia	16.8	2.2	Cross-sectional	173.7	42.02	Low risk
Zurovac [13]	2018	Kenya	53.7	1.8	Cross-sectional	166	25	Low risk
Gallay [14]	2018	Tanzania	59.7	1.12	Cross-sectional	113.9	36	Low risk
Plucinski [12]	2017	Angola	32.8	2.8	Cross-sectional	124	41.51	Low risk
Namuyinga [19]	2017	Malawi	17.6	0.411	Cross-sectional	188.8	47.27	High risk
Pulford [35]	2016	Papua New Guinea	8.4	2.8	Cross-sectional	122.2	47.27	Low risk
Zurovac [16]	2015	Vanuatu	0.285	3.11	Cross-sectional	3.3	0.81	Moderate risk
Landman [36]	2015	Haiti	11	1.27	Cross-sectional	8.4	1.96	Low risk
Steinhardt [37]	2014	Malawi	17.6	0.411	Cross-sectional	188.8	47.27	High risk
Rowe [38]	2009	Angola	32.8	2.8	Cross-sectional	124	41.51	Low risk

Data source: WHO

*Per 1000 population, 2015

**Per 100,000 population, 2015

### The pooled proportion of malaria testing and appropriate treatment by HWs

**Fig. 2:**
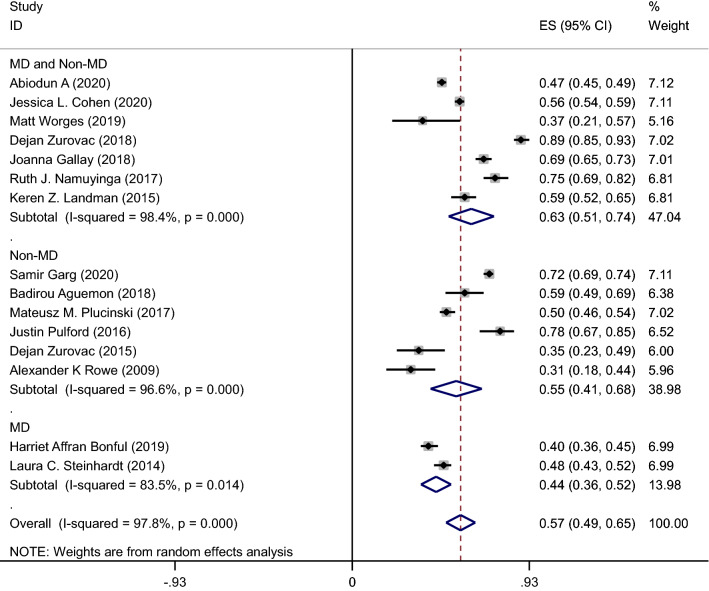
Meta-analysis proportion of malaria testing from suspected cases by HWs cadre

**Fig. 3:**
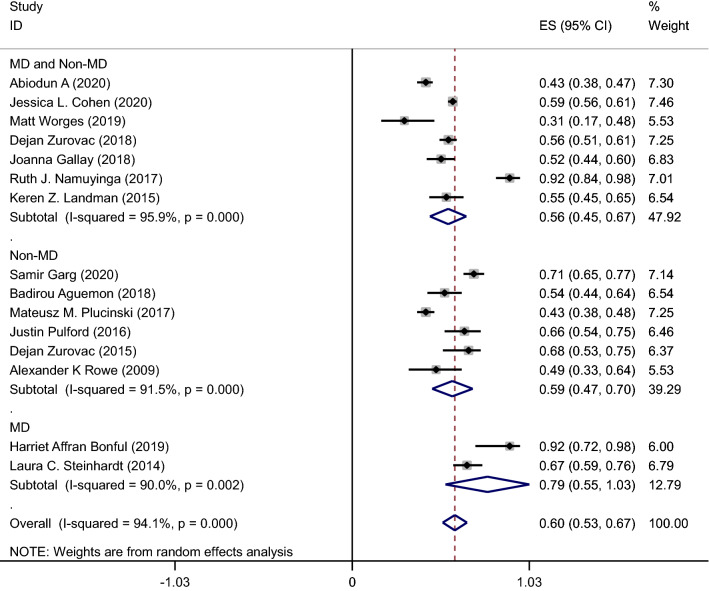
Meta-analysis proportion of HWs appropriate treatment by cadre type

A pooled meta-analysis using random effects for 15 studies indicates an overall malaria testing proportion 57% (95% CI: 49–65%); I^2^ = 97.8%, p < 0.001, and appropriate treatment proportion 60% (95% CI 53–67%, 15 studies); I^2^ = 94.1%, p < 0.001 (Figs. 2 and 3).

### Subgroup analysis by cadre type

#### Proportion of malaria testing

Concerning malaria testing prevalence and subgroup analysis by cadre type, the pooled meta-analysis result using random effects for overall prevalence was 63% (95% CI 51–74%; 7 studies) among MD & Non-MD and 55% (95% CI 41–68%; 6 studies) among Non-MD, 44% (95% CI 36–52%; 7 studies) among MD (Fig. 3).

#### Proportion of appropriate treatment

HWs correct prescribed of the first-line anti-malarial drugs based on positive RDT or microscopy results was used as an appropriate malaria treatment. Appropriate malaria treatment based on test results was as low as 31% % in a Worges study in Zambia [17] to as high as 92% in Bonful in Ghana [18] (Fig. 3).

Figure 3 shows pooled meta-analysis results using random effects for the prevalence of appropriate treatment among various types of HWs cadre. Among HWs cadre, appropriate treatment was higher in MD cadre than in Non-MD and both MD and Non-MD of HWs cadre. Pooled meta-analysis for proportion of appropriate treatment in subgroup analysis by cadre type was 79% (95% CI: 55–100%, 2 studies) in MD, 59% (95% CI: 47–70%, 6 studies) in Non-MD, and was 56% (95% CI: 45–67%, 7 studies) in MD and Non-MD.

### Meta regression analysis

#### Malaria testing

The study was performed a series of multivariable meta-regression analyses to evaluate the potential sources of heterogeneity in malaria testing proportion. The covariates were year of the publication, number of providers, type of cadre, HWs trained for malaria case management, malaria incidence and mortality, total admissions, number of suspected cases admitted in the study period, and availability of diagnostic tests. After a back-step procedure, in which the insignificant variable was deleted after every step, significant or associated variables were retained in the regression model. These seven variables together explained more than 85% of the total variance between studies (Table 4).

**Table 2:** Characteristics of studies included by health worker cadre and availability of anti-malarial drugs and diagnostic tests

Author	Availability of malaria diagnostic tests (%)	Availability of anti-malarial drugs (%)	Health facility (N)	Sample size (9245 HWs)	Cadre	Age of providers (years)	Sex of providers	Trained for diagnosis/treatment
Abiodun [31]	77%	86%	22	154	Clinicians and Nurses	35 (28–43)	57% female	NR
Cohen [22]	NR	NR	6453	7268	MD, Paramedical, CHW, and Nurses	NR	NR	63%
Garg [34]	96%	80%	NR	241	Community HWs	NR	NR	NR
Aguemon [15]	67%	58%	27	93	Community HWs	36 ± 2	73% female	59%
Bonful [18]	NR	NR	NR	82	Medical officers, assistants, nurses	32.2 ± 1.38	61% female	NR
Worges [17]	NR	NR	29	NR	MD, and CHWs	NR	NR	NR
Zurovac [13]	NR	85%	47	182	MD (65%), Nurse	30	NR	40%
Gallay [14]	25%	95.6%	21	187	dispensaries, ADDOs, drug stores, pharmacies	NR	NR	NR
Plucinski [12]	78%	81%	89	212	CHWs	NR	NR	90%
Namuyinga [19]	76%	91%	105	150	Medical assistant, Nurse, attendant	29 Median	73% male	NR
Pulford [35]	NR	NR	NR	265	CHWs (65%) nurse (30%) others (95%)	40 ± 10	58% female	NR
Zurovac [16]	97%	95%	41	67	Nurse (80%), nurse aids and midwives (20%)	40(CI: 38–48)	42% female	60%
Landman [36]	NR	NR	30	115	Not reported	NR	60% female	49%
Steinhardt [37]	4%	81%	107	136	Medical assistant (75%), clinical officer (25%)	36.2 (CI: 21–77)	73% male	83%
Rowe [38]	NR	72.7%	33	93	Nurses, physicians	36 (CI: 21–70)	52% female	60.2%
Total (15 records)	65%	82.5%	7004	9245	Not reported	35.6 (CI: 29– 40)	51% female	55.65%

#### Appropriate treatment

Table 5 showed the results of a multivariable meta-regression analysis of potential sources of heterogeneity in the appropriate malaria treatment. A series of meta-regression analyses was performed to explore the potential sources of the heterogeneity. In the final meta-regression analysis model, appropriateness of the methods, HWs cadre, number of HWs participated in the study, year of the publication, GDP per capita, malaria mortality and morbidity, and availability of anti-malarial drugs together explained more than 83% of the total variance between studies and I^2^ = 33% retained heterogeneity.

**Table 3:** Health workers practice in malaria case management (testing and appropriate treatment)

First author	Total admissions (N)	Suspected Malaria (N)	Malaria patients (N)	Age of patients	Sex of patients (female)	Malaria testing		Appropriate treatment	
						Proportion	95% CI	Proportion	95% CI
Abiodun [31]	3511	1807	431	NR	NR	0.47	0.44–0.49	0.43	0.38–0.47
Cohen [22]	24,756	24,756	7340	23.2	49%	0.56	0.53–0.58	0.59	0.56–0.61
Garg [34]	3087	3087	825	NR	NR	0.72	0.69–0.74	0.71	0.65–0.77
Aguemon [15]	NR	NR	313	NR	52.4%	0.59	0.49–0.69	0.54	0.44–0.64
Bonful [18]	2519	2519	158	3.2	NR	0.4	0.36–0.45	0.92	0.84–0.96
Worges [17]	286	286	39	under 5: 35%	60%	0.37	0.21–0.56	0.30	0.17–0.48
Zurovac [13]	NR	1224	366	NR	NR	0.88	0.85–0.93	0.56	0.51–0.61
Gallay [14]	6391	248	140	NR	NR	0.69	0.64–0.73	0.52	0.435–0.60
Plucinski [12]	1224	790	293	NR	NR	0.49	0.46–0.54	0.43	0.38–0.48
Namuyinga [19]	2096	1427	530	≥ 5: 71%	NR	0.75	0.69–0.82	0.92	0.84–0.98
Pulford [35]	NR	771	122	≤ 15: 64%	53%	0.77	0.67–0.85	0.65	0.54–0.75
Zurovac [16]	226	226	NR	NR	NR	0.35	0.23–0.48	0.68	0.53–0.75
Landman [36]	459	257	11	< 5: 27%	68%	0.59	0.52–0.65	0.55	0.45–0.65
Steinhardt [37]	2019	1747	629	≥ 5: 29%	NR	0.48	0.43–0.52	0.67	0.59–0.76
Rowe [38]	NR	177	59	NR	NR	0.30	0.18–0.43	0.49	0.33–0.64
Total	46,574	39,322	11,256	60%: ≥ 5	57%	0.57	49–65	0.60	53–67

**Table 4:** Results of multivariable meta-regression analysis and the potential source of the heterogeneity of the proportion of malaria testing by health workers (n = 15)

Variables	Coefficient	SE	t	95% CI	P-value
Year of the publication	− 0.036	0.017	− 2.80	− 0.091 to 0.019	0.130
Malaria incidence per 1000	− 0.0012	0.001	− 3.64	− 0.002 to − 0.001	0.036
Malaria mortality per 100,000	0.0044	0.002	1.94	0.001 to 0.0003	0148
number of Health workers	− 0.007	0.001	− 4.10	− 0.01 to − 0.0001	0.026
Health worker cadre	0.211	0.063	3.36	0.01 to 0.411	0.044
Total admissions	0.0004	0.0001	3.33	1.92 to 0.0008	0.045
Total suspected cases	0.0001	0.0005	3.40	0.0001 to 0.0003	0.042
Constant	72.96	35.18	2.07	− 38.99 to 184.92	0.130

I^2^ = 43.16%, Adjusted R^2^ = 85.13%

**Table 5:** Multivariable meta-regression analysis of study variables associated with appropriate malaria treatment (n = 15)

Variables	Coefficient	SE	t	95% CI	P-value
Year of the publication	− 0.009	0.02	− 0.44	− 0.052 to 0.35	0.668
GDP (per capita)	− 0.071	0.043	− 1.63	− 0.18 to 0.40	0.163
Malaria incidence per 1000	− 0.0001	0.003	− 0.51	− 0.001 to 0.006	0.631
Malaria mortality per 100,000	0.0009	0.002	0.03	− 0.0057 to 0.0059	0.964
Number of health worker	− 0.01	− 0.010	− 0.21	− 0.12 to 0.09	0.830
Health worker cadre	− 0.17	0.13	− 1.23	− 0.47 to 0.129	0.242
Availability of anti-malarial drugs	0.124	0.35	0.35	− 0.79 to 1.03	0.740
Appropriateness of sampling and methods (risk of bias)	0.200	0.085	2.36	− 0.01 to 0.419	0.066
Constant	0.368	0.36	1.00	− 0.58 to 1.31	0.365

I^2^ = 33.4%

Adjusted R-squared (R^2^) = 83.43%

## Discussion

This study is one of the rare meta-analyses and meta-regression demonstrating HWs performance in malaria case management by evaluating testing and treating measures including the pooled proportions estimate of malaria testing among febrile patients compatible with suspected malaria, and appropriate malaria treatment. The study found some strengths however major deficiencies in HWs practice and also system readiness and clinical practice which severely compromise the quality of service delivery for confirmed and suspected malaria patients. However, there are limited studies based on systematic reviews, to measure the practice of HWs in case management of malaria patients by considering significant outcome measures.

The findings revealed that the pooled proportion estimate of malaria testing (57%; 95% CI: 49–65%); and appropriate treatment based on RDT or microscopy results (60%; 95% CI: 53–67%); were generally low among HWs. Regarding subgroup analysis of malaria testing and treating by HWs cadre, MD providers had the highest proportion of appropriate treatment (79%) while the lowest malaria testing and screening proportions from suspected cases was seen also among MD cadre (44%). The highest proportion of appropriate malaria treatment was found in Namuyinga [19] (Malawi) and Bonful [18] (Ghana) studies with 92%, while the lowest percentage (30.5%) was in Worges study in Zambia [17].

The results of multivariable meta-regression analysis explored the effects of variables including HW cadre, number of HWs, malaria incidence, malaria mortality, total admissions and malaria suspected cases, and year of the publication on pooled proportion estimate of conducting malaria testing form suspected cases, as the potential sources of the heterogeneity. Likewise, appropriateness of methods and sampling, HWs cadre and numbers, GDP per capita, malaria incidence and mortality, and availability of anti-malarial drugs were the potential sources of the heterogeneity in the pooled proportion estimating of appropriate treatment by HWs. Risk of bias assessment found that articles with the appropriateness of sampling and methods, using appropriate data collection methods for example assessed HWs practice by valid and reliable methods including direct view, performed blood samples of the patients, interviews with HWs, and/or having a gold standard for evaluating HWs practice estimated actual parameter estimation in the percentage of appropriate malaria treatment, due to the robust and unbiased methods and sampling strategies. On the other hand, studies with a high risk of bias and inappropriateness of methods overestimate the prevalence/proportion of appropriate malaria treatment by HWs.

Treatment with first-line anti-malarial drugs and ACT (not only prescribing any anti-malarial) are critical to preventing the progression of malaria to severe disease and lethal outcome. The case fatality rate of untreated severe malaria has been estimated 13–21% [20]. Although, ACT became the WHO recommended first-line treatment for malaria in 2006 [21], stocking and availability of ACT was incomplete in some of the studies included.

There are a number of reasons why HWs may not be providing appropriate anti-malarial drugs and malaria diagnostic tests to patients with a malaria diagnosis in the included studies. Testing may be incomplete because HFs lack a licensed microscopist or laboratory technician, because HWs symptom-based identification, or high patient volumes and/or high out-of-pocket costs of the diagnostic tests [22]. In sub-Saharan African countries or resource limited settings, HWs may not be receiving appropriate anti-malarial drugs due to unavailability of appropriate and first-line anti-malarial drugs, because of provider or patient preference for alternative medications, or because of high out-of-pocket costs for these prescriptions [22–24]. Some evidence have found that malaria patients continue to face out-of-pocket costs for health services and commodities that are intended to be provided free in settings similar to the study countries [25–27].

Regarding the availability of anti-malarial drugs and diagnostic tests, the study results showed that an overall proportion of 82.5 and 65%, respectively. Considering the high diagnostic accuracy of RDT (99% sensitivity vs 90% specificity) for both falciparum and non-falciparum malaria, the availability of RDT provide prompt diagnosis and treatment for malaria cases and also prevent malaria misdiagnosis [28]. So, HWs can appropriately diagnose and treat malaria using RDT in resource limited settings [29]. It seems that the most of HWs were able to identify that fever required testing with an RDT. However, findings have been showed almost half of HWs did not obtain a fever history among patients who did not spontaneously report one or did not record a temperature, which prevents accurate estimates of the number of suspected cases who receive an RDT [30].

In the absence or low availability of RDT, the introduction of the quality assurance system for malaria microscopy, prioritization of microscopy for febrile inpatient management, and increased health facilities availability of malaria RDTs focusing on outpatient malaria screening should be the programmatic and organizational priorities targeting improved diagnostic services in the various settings [31].

This study findings provided a comprehensive evidence for evaluating HWs readiness and practice and also weakness and strengthens in the appropriate malaria case management. HWs appropriate malaria case management is a key measure to minimize malaria mortality and morbidity in both low and high malaria transmission settings and to promote malaria control programmes. Moreover, HWs readiness and practice are considered authentic evidence to measure the health system readiness regarding malaria control programme milestones and to issue malaria elimination certification [8]. Likewise, malaria testing and screening from febrile outpatients compatible with suspected malaria can prevent lethal outcomes and increased timely and prompt case finding and diagnosis especially in high transmission settings. In low transmission settings, it could timely identify imported cases and provide credible evidence to measure prevention of re-establishment of malaria and also elimination criteria [32].

Malaria elimination programmes are rigorous and included appropriate case management, testing all febrile cases in all ages throughout the year, treating all confirmed malaria patients with ACT, case investigation to determine whether the case was locally acquired, and reinforcing malaria knowledge and practices despite the declining risk [33]. Currently, a study provided core recommendations for malaria elimination including adapting surveillance systems, deeper researches with role-playing technique, reinforcing system readiness (vigilance) and sensitivity in case management, and mobilization to become more granular and quick to respond to malaria testing and treating [8].

## Limitations

This is the first systematic review, meta-analysis, and meta-regression analysis indicating the pooled prevalence estimate of malaria testing from suspected cases and malaria appropriate treatment by various HWs cadre. However, the present study had limitations. The main concern was including different studies from the different populations (countries) with various malaria transmission settings for estimating the pooled prevalence of appropriate malaria testing and treatment. To solve this problem, we measured and involved the potential effects of population size, malaria transmission setting (malaria mortality and morbidity), GDP of countries, and also the appropriateness of sampling and methods (risk of bias) using multivariable meta-regression analysis to explore the potential source of heterogeneity.

## Conclusion

This systematic review and meta-analysis findings demonstrated the pooled proportion estimate of appropriate treatment (60%; 95% CI: 53–67%); and malaria testing (57%; 95% CI: 49–65%). HWs compliance to appropriate malaria case management were generally low while no study was found in countries in eliminating phase.

In the final multivariable meta-regression analysis model, appropriateness of sampling and methods, HWs cadre and numbers, malaria mortality and morbidity, total admissions and malaria suspected cases, availability of antimalarial drugs, GDP per capita, and year of the study conducted explained almost 85.0% of the total variance between studies and potential sources of the heterogeneity in the proportion of malaria testing and appropriate treatment. Studies with the inappropriateness methods and risk of bias could be overestimating the actual prevalence of malaria appropriate testing and treating.

## Recommendations

Establishments of the effective supply chain for HWs, providing in-service training programs, quality-assured diagnostics, ongoing support for HWs to deliver care conferring to the guidelines, and close monitoring of the systems readiness and clinical practices will ultimately determine the attainment of the policy translation continue the importance of practice and quality of appropriate malaria case-management are required.

Continued progress in the quality of care for malaria requires deeper research into the lingering obstacles and facilitators of appropriate malaria case management especially in eliminating settings where they are progressing for malaria elimination certification and by emphasizing that no studies have been conducted in these countries.

Strategies that focus on improving the identification of acute febrile diseases especially with similar clinical and epidemiological features with malaria should be highly promoted. Moreover, role-playing and implementation research are recommended to recognize the underlying factors that can affects to the success of large-scale interventions in the appropriate malaria case management and progress measures for malaria elimination certification.

## Data Availability

The datasets generated and/or analysed during the current study are available from the corresponding author on reasonable request.

## Abbreviations

ACT: Artemisinin-based combination therapy
CI: Confidence interval
CHWs: Community health workers
GDP: Gross domestic product
MD: Medical doctor
HFs: Health facilities
HWs: Health workers
RDTs: Rapid diagnostic tests
WHO: World Health Organization
